# The hidden IVUS effect: a meta-epidemiological analysis of control-arm optimization and treatment-effect attenuation in contemporary IVUS-guided PCI trials

**DOI:** 10.1093/ehjopen/oeag111

**Published:** 2026-06-30

**Authors:** Giuseppe Panuccio, Youssef S Abdelwahed, Ulf Landmesser, Salvatore De Rosa, Daniele Torella

**Affiliations:** Department of Experimental and Clinical Medicine, Magna Graecia University, Viale Europa, 88100 Catanzaro, Italy; Department of Cardiology, Angiology and Intensive Care Medicine, Deutsches Herzzentrum der Charite, Hindenburgdamm 30, 12203 Berlin, Germany; Cardiovascular Research Center, Magna Graecia University, Viale Europa, 88100 Catanzaro, Italy; Department of Cardiology, Angiology and Intensive Care Medicine, Deutsches Herzzentrum der Charite, Hindenburgdamm 30, 12203 Berlin, Germany; Department of Cardiology, Angiology and Intensive Care Medicine, Deutsches Herzzentrum der Charite, Hindenburgdamm 30, 12203 Berlin, Germany; DZHK (German Centre for Cardiovascular Research), Potsdamer Straße 58, 10785 Berlin, Germany; Berlin Institute of Health (BIH), Anna-Louisa-Karsch-Straße 2, 10178 Berlin, Germany; Department of Medical and Surgical Sciences, Magna Grecia University, Viale Europa, 88100 Catanzaro, Italy; Cardiovascular Research Center, Magna Graecia University, Viale Europa, 88100 Catanzaro, Italy; Department of Medical and Surgical Sciences, Magna Grecia University, Viale Europa, 88100 Catanzaro, Italy

**Keywords:** Intravascular ultrasound, Percutaneous coronary intervention, Coronary artery disease, Coronary Imaging, Precision Medicine

**Figure 1:**
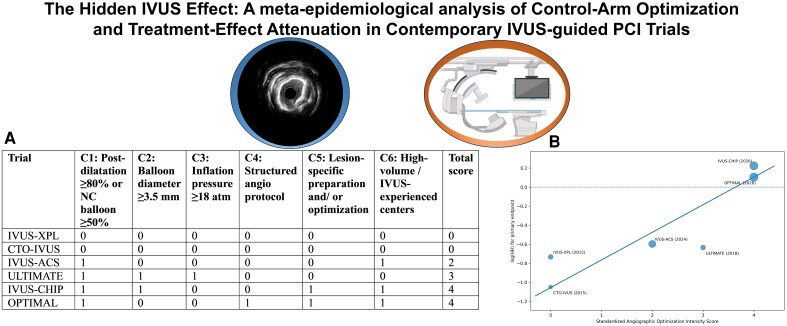
Trials and respective domains of the angiographic optimization intensity index (*A*) and meta-regression analysis for the interaction between the index and the log(HR) for the trial primary endpoint. HR, hazard ratio.

The publication of recent randomized controlled trials (RCTs) of intravascular ultrasound (IVUS)-guided percutaneous coronary intervention (PCI) has raised discussion among the interventional cardiology community. Several RCTs showed improved clinical outcomes with IVUS guidance, whereas the recent IVUS CHIP and OPTIMAL did not demonstrate significant superiority of IVUS guidance over angiography-guided PCI.^1,2^ This apparent contradiction raises a question: is the incremental benefit of IVUS driven only by the imaging strategy, or also by the quality of the angiography-guided comparator? Several comments have been made on these findings, but we sought to formally test these hypotheses. Therefore, we aimed to assess whether angiography-guided control-arm optimization was associated with attenuation of the apparent incremental benefit of IVUS-guided PCI across contemporary RCTs. We hypothesized that neutral contemporary IVUS trials may partly reflect a ‘hidden IVUS effect’, whereby angiography-guided control arms progressively incorporate IVUS-derived procedural principles. These include more aggressive lesion preparation, non-compliant balloon use, high-pressure post-dilatation, larger device selection, anatomy-specific optimization, and operator expertise, which may have influenced the quality of the angiography-guided arms. Accordingly, we sought to detect and quantify a ‘hidden IVUS effect’ across contemporary IVUS RCTs, performing a meta-epidemiological analysis at the trial level of RCTs comparing IVUS-guided vs. angiography-guided PCI with available clinical hazard ratios (HRs) for the primary endpoint. Six RCTs were included: IVUS-XPL, CTO-IVUS, ULTIMATE, IVUS-ACS, IVUS-CHIP, and OPTIMAL.^1–6^ Trials without random allocation to imaging vs. angiography guidance, or powered for angiographic rather than clinical endpoints, were excluded from this analysis. Accordingly, major RCTs like RENOVATE-COMPLEX-PCI were excluded since the imaging-guided arm allowed both IVUS and OCT. For each trial, we extracted the HRs for the primary endpoint and procedural markers of optimization in the angiography-guided arm. Accordingly, we constructed a pre-specified Angiographic Optimization Intensity Index. One point was assigned for each of the following control-arm domains: 1) post-dilation ≥80% or, when unavailable, non-compliant balloon use ≥50%; 2) maximum balloon diameter ≥3.5 mm; 3) maximum balloon inflation pressure ≥18 ATM; 4) explicit structured angiographic optimization protocol; 5) lesion-specific preparation and/or optimization, for example, cutting/scoring balloon, intravascular lithotripsy, rotational/orbital atherectomy, or anatomy-specific optimization technique when applicable ≥80%; 6) trial-level restriction to high-volume or IVUS-experienced centers/operators (*Figure 1A*). Log-transformed HRs were modeled using weighted meta-regression, with inverse-variance weighting. Standard errors were derived from the published 95% confidence intervals. The included trials varied substantially in the angiography-guided comparator. For example, in CTO-IVUS, the angiography-guided arm had high-pressure post-stent dilation in 41.3% of the cases, maximum post-stent balloon pressure of 13.8 ± 3.8 ATM, and mean stent diameter of 2.85 ± 0.41. Conversely, IVUS-CHIP and OPTIMAL had highly optimized angiography-guided arms and enrolled high-volume or IVUS-experienced centres. In weighted meta-regression analysis, a higher Angiographic Optimization Intensity Index was associated with attenuation of the apparent benefit of intravascular imaging. Each 1-point increase in this index was associated with a higher log(HR) for the primary endpoint, indicating a shift of the treatment effect towards neutrality (β = 0.290 per point; SE = 0.067; *P* = 0.012; weighted *R*^2^= 0.82; *Figure 1B*). These findings should not be considered as evidence that angiography-guided PCI is equivalent to IVUS-guided PCI. Conversely, they suggest that an angiographic comparator arm, which benefit from an ‘IVUS-like’ approach, based on adequate post-dilation, adequate device sizing, and a high experience of IVUS-guided PCI, may attenuate treatment differences. In this context, neutral IVUS trials should not automatically be interpreted as evidence against IVUS, but rather as evidence that there is a ‘hidden IVUS effect’ that may have shaped the angiographic arm comparators towards a higher quality PCI, which is closer to the IVUS-guided procedures. These findings suggest that intravascular imaging may have shaped contemporary interventional practice even when not directly used for procedural guidance. Moreover, in the IVUS CHIP trial, only around 50% of included patients met the pre-specified IVUS stent optimization criteria. Therefore, these findings support the need for broader training in imaging-derived principles to obtain direct and indirect effects that will improve patients’ care. These findings have limitations, including aggregate trial-level data, a small number of studies, and the use of a non-validated optimization index. Nevertheless, this analysis highlights an important design issue for future PCI trials. In conclusion, the apparent benefit of IVUS-guided PCI may be attenuated when angiography-guided PCI incorporates IVUS-derived optimization practices. Comparator arms should be reported with the same granularity as imaging arms, including post-dilatation rate, non-compliant balloon use, balloon and stent dimension, inflation pressures, lesion preparation, use of proximal optimization technique, operator imaging expertise, and achievement of angiographic optimization criteria. Rather than asking only if IVUS works, future trials should also ask what exactly IVUS is being compared against.
